# Microvascular Dysfunction in Skeletal Muscle Precedes Myocardial Vascular Changes in Diabetic Cardiomyopathy: Sex-Dependent Differences

**DOI:** 10.3389/fcvm.2022.886687

**Published:** 2022-05-18

**Authors:** Sadi Loai, Xuetao Sun, Mansoor Husain, Michael A. Laflamme, Herman Yeger, Sara S. Nunes, Hai-Ling Margaret Cheng

**Affiliations:** ^1^Institute of Biomedical Engineering, University of Toronto, Toronto, ON, Canada; ^2^Translational Biology and Engineering Program, Ted Rogers Centre for Heart Research, Toronto, ON, Canada; ^3^Toronto General Hospital Research Institute, University Health Network, Toronto, ON, Canada; ^4^Peter Munk Cardiac Centre, University Health Network, Toronto, ON, Canada; ^5^Ted Rogers Centre for Heart Research, Toronto, ON, Canada; ^6^McEwen Stem Cell Institute, University Health Network, Toronto, ON, Canada; ^7^Laboratory of Medicine and Pathobiology, University of Toronto, Toronto, ON, Canada; ^8^Program in Developmental and Stem Cell Biology, The Hospital for Sick Children, Toronto, ON, Canada; ^9^The Edward S. Rogers Sr. Department of Electrical and Computer Engineering, University of Toronto, Toronto, ON, Canada

**Keywords:** heart failure, microvascular dysfunction, skeletal muscle, sex differences, type II diabetes, preserved ejection fraction (HFpEF), histopathology

## Abstract

**Aim:**

To uncover sex-related microvascular abnormalities that underlie the early presentation of reduced perfusion in leg skeletal muscle in a type II rat model of diabetic cardiomyopathy.

**Methods and Results:**

Diabetes was induced using a non-obese, diet-based, low-dose streptozotocin model in adult female (18 diabetic, 9 control) and male rats (29 diabetic, 11 control). Time-course monitoring over 12 months following diabetes induction was performed using echocardiography, treadmill exercise, photoacoustic imaging, flow-mediated dilation (FMD), histopathology, and immunohistochemistry. Diabetic rats maintained normal weights. Hypertension appeared late in both diabetic males (7 months) and females (10 months), while only diabetic males had elevated cholesterol (7 months). On echocardiography, all diabetic animals maintained normal ejection fraction and exhibited diastolic dysfunction, mild systolic dysfunction, and a slightly enlarged left ventricle. Exercise tolerance declined progressively and early in males (4 months), later in females (8 months); FMD showed lower baseline femoral arterial flow but unchanged reactivity in both sexes (5 months); and photoacoustic imaging showed lower tissue oxygen saturation in the legs of diabetic males (4 months) and diabetic females (10 months). Myocardial perfusion was normal in both sexes. Histopathology at the final timepoint of Month 10 (males) and Month 12 (females) revealed that myocardial microvasculature was normal in both vessel density and structure, thus explaining normal perfusion on imaging. However, leg muscle microvasculature exhibited perivascular smooth muscle thickening around small arterioles in diabetic females and around large arterioles in diabetic males, explaining the depressed readings on photoacoustic and FMD. Histology also confirmed the absence of commonly reported HFpEF markers, including microvessel rarefaction, myocardial fibrosis, and left ventricular hypertrophy.

**Conclusion:**

Exercise intolerance manifesting early in the progression of diabetic cardiomyopathy can be attributed to decreased perfusion to the leg skeletal muscle due to perivascular smooth muscle thickening around small arterioles in females and large arterioles in males. This microvascular abnormality was absent in the myocardium, where perfusion levels remained normal throughout the study. We conclude that although skeletal muscle microvascular dysfunction of the vasculature presents at different levels depending on sex, it consistently presents early in both sexes prior to overt cardiac changes such as rarefaction, fibrosis, or hypertrophy.

## Introduction

In the past decade, microvascular dysfunction (mvD) has been increasingly recognized as an early hallmark in the progression of both heart failure with reduced (HFrEF) and preserved ejection fraction (HFpEF) due to its association with multiple comorbidities—e.g. diabetes, hypertension, and obesity (1–3). The phenomenon is posited to be a consequence of chronic low-grade inflammation (4) and describes, on the whole, the impaired regulation of blood flow in small blood vessels, encompassing structural changes such as vessel rarefaction or functional changes such as abnormal vasodilation and constriction (5). Most studies on mvD in the heart failure setting have focused on the coronary microcirculation, with recent reports demonstrating the prevalence of coronary mvD in diabetic patients (6) and, more broadly, the power of coronary mvD as an independent prognostic predictor in HFpEF patients (7). Peripheral skeletal muscle vascular alterations, on the other hand, remain poorly understood, particularly in the HFpEF phenotype, despite its association to the symptom of exercise intolerance seen in all heart failure patients. An emerging literature on extra-myocardial changes is beginning to cast light on leg skeletal muscle vascular abnormalities in heart failure: perfusion deficits manifest early (8) and capillary loss underlies impaired blood flow response to leg muscle contractions (9). However, much remains unknown, such as the underlying cause of the perfusion deficit or the temporality of changes in peripheral skeletal muscle vessels compared to myocardial vasculature.

In this study, we expand on our previously published preclinical study of diabetic cardiomyopathy that mapped out disease onset and progression in a type II diabetes (T2D) model in male rats (8). The previous study revealed early perfusion deficits only in the leg skeletal muscle on photoacoustic imaging but not in the heart, deficits that presented circa the onset of exercise intolerance but before the onset of hypertension, cardiac fibrosis, microvascular rarefaction, or other established clinical markers. To determine the root cause underlying this perfusion deficit in the leg muscle, we undertook an in-depth histopathological analysis of the myocardium and skeletal muscle. As a secondary aim, we included female rats to uncover potential sex-related differences that emerge at the vascular level, as men and women with T2D do present clinically distinct vascular complications (10, 11). Our immediate goal was to attain a deeper understanding of the sex-dependent pathology of mvD in diabetic cardiomyopathy and the temporal relationship between mvD in skeletal muscle vs. myocardial tissue. Ultimately, our findings would uncover diagnostic biomarkers during the early progression of diabetic cardiomyopathy.

## Materials and Methods

### Animals and Disease Induction

**Figure 1:**
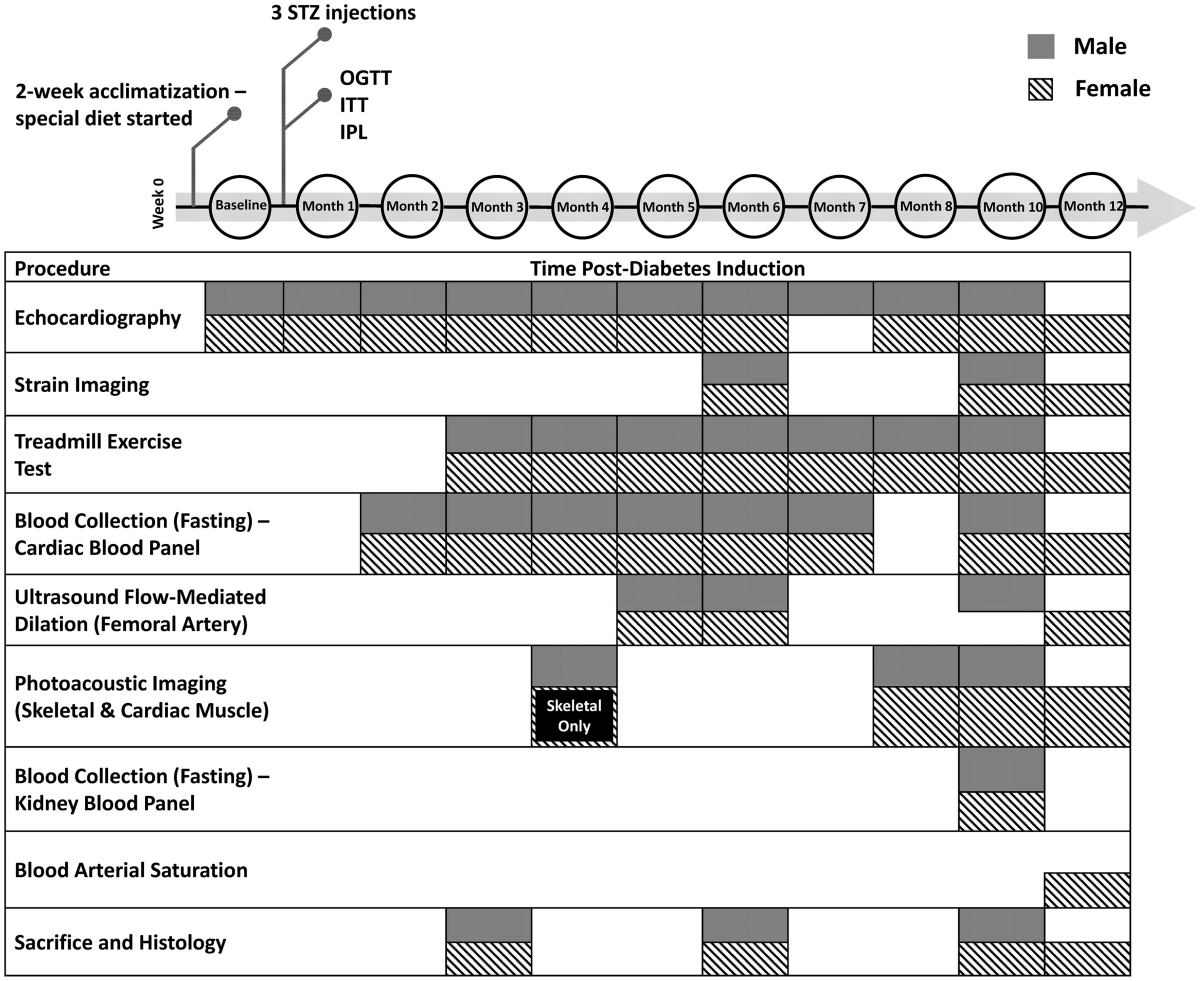
Timeline of diabetes induction and post-induction monitoring and assessment. Additional measurements not shown include weekly weight, non-fasting blood glucose, and monthly blood pressure measurements. STZ, streptozotocin; OGTT, oral glucose tolerance test; ITT, insulin tolerance test; IPL, insulin plasma level

This study was approved by the University of Toronto's Animal Care Committee (protocol #20012191). All procedures were conducted in accordance with the Canadian Council on Animal Care. The full experimental timeline and all procedures performed are illustrated in Figure 1.

Six- to seven-week-old female Sprague Dawley rats (Charles River, Quebec, Canada) were housed two per cage with a 12:12-h light-dark cycle and constant room temperature (23°C ± 1°C). Rats were divided into two groups: diabetic female rats (*N* = 18) on a high-fat/high-sucrose diet containing 20.14wt% sucrose and 20.68wt% lard (D12451, Research Diets Inc., USA) and control female rats (*N* = 9) on a calorie-matched control diet (D12450K, Research Diets Inc., USA). All rats were fed ad libitum for the full duration of the study and had free access to water. After 2 weeks of acclimatization, diabetic rats were fasted overnight and injected intraperitoneally (IP) with a low dose of streptozotocin (STZ, 30 mg/kg, Millipore Sigma, 572201), dissolved in a 0.1M citrate buffer, pH 4.5. The control group underwent the same procedure but were given a vehicle injection (citrate buffer). STZ injections were repeated a second time 2 weeks later in all 18 animals, 13 rats required a third injection 2 weeks after the second injection to chronically stress Langerhans islets cells and induce a state of frank diabetes. Following the 8-week diabetes induction period, the diabetic cohort was monitored for 1 month to confirm the presence of type II diabetes. Diabetes was defined as having a non-fasting blood glucose level ≥ 15 mmol/L and a fasting blood glucose level ≥ 10 mmol/L. All timepoints mentioned in this study refer to months post-induction of diabetes. To ensure diabetes was maintained throughout the study, non-fasting blood glucose measurements (glucometer, Accu-Chek Aviva) from a tail vein prick were collected on a weekly basis. A breakdown of the male cohort can be found in our previously published study (8).

### Confirmation of Glucose and Insulin Intolerance

Immediately following the 8-week diabetes induction period, all rats underwent an oral glucose tolerance test (OGTT) to confirm glucose intolerance in diabetic rats. Animals were fasted overnight and then given an oral dose of glucose solution (BioShop Canada Inc.) at 1 g/kg *via* gavage. Blood glucose was measured over 2 h, prior to gavage and at 30-min intervals post-glucose consumption.

One week following the OGTT, all rats underwent an insulin tolerance test (ITT) to assess diabetes-related insulin resistance. Animals were fasted for 6 h and injected IP with insulin solution (0.75 U/kg, Sigma-Aldrich, I9278). Blood glucose was measured prior to injection, at 15-min intervals post-injection for the 1st hour, and 30-min intervals for the 2nd hour.

One-month post-induction, blood plasma insulin levels were tested in all rats to confirm insulin resistance in diabetic rats. Animals were fasted overnight, and blood from the saphenous vein was collected in lithium-heparin tubes. Tubes were centrifuged for 8 min at 8,000 relative centrifugal force (rcf) and blood plasma was separated from the samples. A rat insulin Sandwich ELISA kit (Sigma-Aldrich, RAB0904) was used to determine insulin levels in the plasma samples. Briefly, insulin molecules from blood plasma were captured in a microtiter plate that was coated with monoclonal anti-rat insulin antibodies. Biotinylated polyclonal antibodies bound to the captured insulin and any unbound material was washed away. Horseradish peroxidase was then bound to the immobilized antibodies and quantification of enzyme activity was measured spectrophotometrically at 450 nm. The increase in absorbance was directly proportional to the amount of captured insulin in each sample. All samples were tested in duplicates. A homeostatic model assessment of insulin resistance (HOMA-IR) was then calculated to assess insulin resistance (12).

### Blood Chemistry

A cardiac blood panel was run monthly over Months 2 to 7 post-induction and then again at Month 10 and 12 post-induction. Levels of high-density lipoprotein (HDL)-cholesterol, low-density lipoprotein (LDL)-cholesterol, total cholesterol, triglyceride, and fasting blood glucose were measured. A kidney blood panel was run at Month 10 post-induction to measure levels of creatinine, albumin, and total protein. Animals were fasted overnight, and blood from the saphenous vein was collected in lithium-heparin tubes. Tubes were centrifuged for 8 min at 8000 rcf and blood plasma was separated from the samples. Blood plasma was analyzed with a Beckman Coulter analyzer at The Center for Phenogenomics, Toronto, Canada. Briefly, plasma samples were aliquoted into reaction vessels and diluted with reagents optimized for the metabolite being measured. The mixture was passed through a colorimeter for absorbance measurements and the analyte concentration was determined.

### Blood Pressure Measurement

Non-invasive measurements of heart rate, systolic, diastolic and mean blood pressure (BP) were taken monthly in conscious rats using a CODA Monitor (Kent Scientific, USA). Rats were placed into holders and measurements were obtained from a tail cuff. An average of three measurements were collected from each rat in a quiet environment.

### Treadmill Exercise Tolerance Testing

Exercise tolerance, as described previously (8), was determined by assessing the change in running distance on a treadmill, with baseline measurements beginning at Month 3 post-induction (Table 1). In brief, rats were placed on an exercise regime where the treadmill (Maze Engineers, USA) inclination was set at 12° and the speed started at a slow pace of 5 m/min for 2 min. The speed was increased to 10 m/min for another 2 min, followed by a 2 m/min increment every 2 min up to a maximum speed of 20 m/min or until the rat reached exhaustion. Exhaustion was defined as either the rat touching an electrical stimulus grid seven times with a minimum contact period of 1 sec, or the rat failing to continue running within 7 sec of full contact with the grid. The regime ended once the rat reached exhaustion or, in the absence of exhaustion, 20 min of exercise had elapsed. All rats were acclimatized to the treadmill regime for several days prior to data collection.

**Table 1:** Absolute treadmill running distance at month 3 post-diabetes induction.

	**Running Distance (m)**
**Male Control**	97.9 ± 13.9
**Male Diabetic**	95.4 ± 18.7
**Female Control**	282.3 ± 13.6
**Female Diabetic**	205.8 ± 27.8

### *In-vivo* Ultrasound and Photoacoustic Imaging

A high-frequency ultrasound and photoacoustic imaging system (Vevo 3100/LAZR-X, FUJIFILM VisualSonics Inc., Toronto) equipped with transducers of different operating frequencies was used. An MS201 transducer (12–15MHz) was used for all transthoracic cardiac and photoacoustic imaging. An MX400 transducer (30 MHz) was used for all femoral artery and skeletal muscle photoacoustic imaging. Anesthesia was induced on 5% isoflurane (in 100% oxygen) delivered through a facemask for about 1 min and maintained on 1.5–2.0% isoflurane throughout imaging. The paws were taped to electrodes for electrocardiogram (ECG) signal monitoring. Body temperature was maintained at 37°C and monitored with a rectal thermal probe. Isoflurane was chosen among other anesthetics due to its fast-acting and short-lasting nature, and it is the preferred anesthetic for echocardiography for minimizing cardiorespiratory depression effects (13). Imaging sessions were kept under 20 min to avoid prolonged exposure to isoflurane and variability in heart rates between rodents. A full protocol of the imaging technique and analysis for echocardiography, photoacoustic imaging, and femoral artery flow-mediated dilation (FMD) can be found in our previously published study (8).

Strain Imaging was also performed at Month 6 and 10/12 post-induction. Analysis was conducted on B-mode CINE loops of the left ventricle (LV) in the long-axis view on the VevoLab Strain Analysis software (FUJIFILM VisualSonics Inc., Toronto). The endocardium and epicardium of the LV was manually traced for each animal prior to automatic segmentation and analysis. Global longitudinal strain, endocardium longitudinal strain, and radial strain were reported.

### Arterial Oxygen Saturation

At Month 12 post-induction, arterial oxygen saturation was measured prior to sacrifice in female control and diabetic rats. Animals were induced with 5% isoflurane delivered through a facemask for about 1 min and maintained on 1.5–2.0% isoflurane. Arterial oxygen saturation was measured *via* a MouseOx Plus unit (STTARR Life Sciences Corp. USA), using a dedicated rat foot clip sensor.

### Histopathology and Immunohistochemistry

At the sacrificial timepoints indicated in Figure 1, rats were euthanized on 4% isoflurane and 1 mL 10% potassium chloride injection into the right ventricle to stop the heart in end-diastole. The heart was perfused with saline and harvested. Tissues were fixed overnight in formalin and embedded in paraffin. The heart was sectioned in the transverse plane into three parts (atria, ventricles, and apex). Sectioned slices of the heart (5 μm) were stained with haematoxylin and eosin (H&E) for cross-sectional area (CSA), picrosirius red (PSR) for fibrosis, CD31 (Abcam, EPR17259) for capillary quantification, and alpha-smooth muscle actin (αSMA) (Abcam, ab124964) for vascular smooth muscle wall thickening and myofibroblast deposition. The gastrocnemius skeletal muscle of the leg was stained with anti-CD31 and αSMA.

All sections were imaged on a Leica DMi8 inverted epifluorescence microscope using brightfield imaging. For each section, two to four slices, with five to ten images per slice uniformly covering all areas, were captured at 10x magnification. Myocardial and skeletal muscle capillary density was quantified using in-house software developed in Python, utilizing OpenCV library for automatic shape detection and the Canny algorithm for edge detection, with consistent thresholding across all images and RGB detection. For assessment of myocardial interstitial fibrosis, areas of periadventitial collagen surrounding vessels were avoided. Images were quantified for collagen and αSMA content on ImageJ software, utilizing RGB stack images and consistent segmentation thresholds across all images (14). Cardiomyocyte CSA was assessed on ImageJ by measuring the individual CSA of 50–100 myocytes from the anterior and posterior LV wall.

Immunofluorescence staining was performed on cardiac and skeletal tissues. Staining for CD31, αSMA (Abcam, ab220179), and DAPI (Abcam, ab228549) was conducted to identify arterioles and capillaries. CD31 was conjugated to Alexa Fluor 647 (Fisher, A-31571); αSMA was conjugated to Alexa Fluor 488 (Fisher, A-21206). Fluorescence imaging captured at 20x magnification was performed on an Olympus FLUOVIEW FV3000 confocal laser scanning microscope. Twenty to 30 arterioles with diameters in the 10–25 μm range were identified for each tissue section; the ratio αSMA/CD31, representing the perivascular smooth muscle surface area relative to endothelium, was averaged across all arterioles. Quantification was conducted using ImageJ; each individual vessel was separated into RGB channels, and surface area coverage was measured for the smooth muscle wall and endothelium independently.

### Statistical Analysis

All data is represented as mean ± standard error of the mean (SEM). Data was analyzed in Matlab (version R2020a) using a three-way analysis of variance (ANOVA), where time, diet, and sex were independent variables. If a significant group effect was uncovered, Fisher's *post-hoc* analysis was performed for multiple comparisons. In all cases, significance was reported at a *p*-value of 5%.

## Results

### Skeletal Muscle Microvasculature Dysfunction

**Figure 2:**
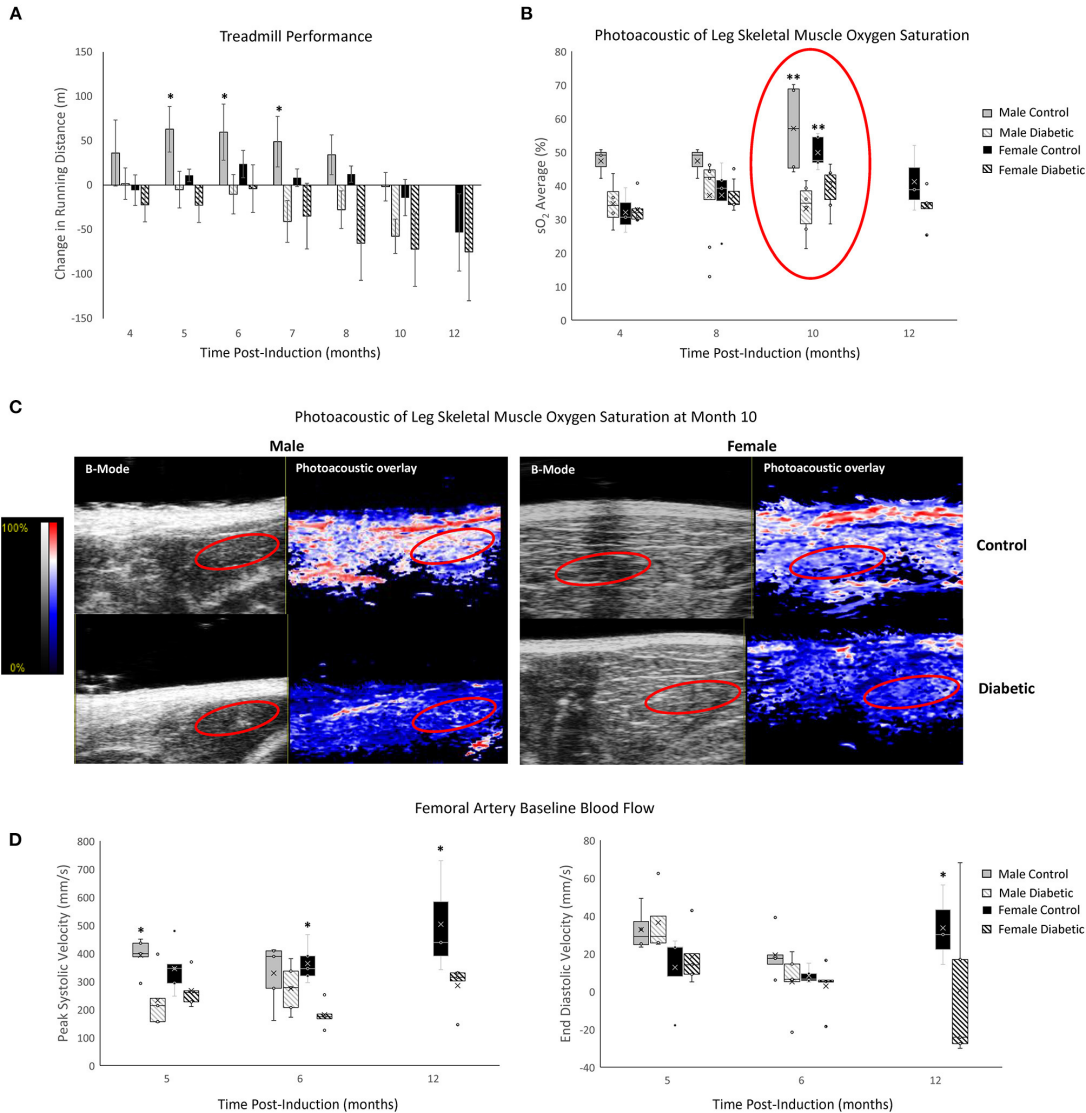
Non-invasive evaluation of skeletal muscle microvasculature dysfunction. **(A)** Treadmill exercise tolerance testing. The change in running distance relative to Month 3 in individual rats is averaged for each cohort. Significance between male or female control/diabetics across all timepoints was found at *p* = 0.0024 and *p* = 0.0353, respectively. Animal numbers at 3 and 4 months (*n* = 8 control male, *n* = 17 diabetic male, *n* = 8 control female, and *n* = 12 diabetic female), 5 and 6 months (*n* = 8 control male, *n* = 12 diabetic male, *n* = 7 control female, and *n* = 12 diabetic female), 7 and 8 months (*n* = 6 control male, *n* = 9 diabetic male, *n* = 5 control female, and *n* = 9 diabetic female), 10 months (*n* = 6 control male, *n* = 8 diabetic male, *n* = 5 control female, and *n* = 9 diabetic female), and 12 months (*n* = 3 control female, and *n* = 5 diabetic female). **(B)** Tissue oxygen saturation in leg skeletal muscle from photoacoustic imaging at 4 months (*n* = 3 control male, *n* = 4 diabetic male, *n* = 3 control female, and *n* = 6 diabetic female), 8 months (*n* = 3 control male, *n* = 8 diabetic male, *n* = 5 control female, and *n* = 7 diabetic female), 10 months (*n* = 4 control male, *n* = 6 diabetic male, *n* = 5 control female, and *n* = 6 diabetic female), and 12 months (*n* = 3 control female and *n* = 5 diabetic female) post-induction. **(C)** Photoacoustic ultrasound images of skeletal muscle at 10 months post-induction. Greyscale images are B-Mode images, and colormaps are the photoacoustic tissue oxygenation saturation overlay. **(D)** Baseline measurements from the femoral artery for peak systolic velocity and end-diastolic velocity measured at month 5 (*n* = 5 for each of control male, diabetic male, control female, and diabetic female), month 6 (*n* = 5 for each of control male, diabetic male, control female, and diabetic female), and month 12 (*n* = 3 control female and *n* = 5 diabetic female) post-induction. Boxplot mean value is represented with the symbol “X.” Significance at individual timepoints between control and diabetic cohorts of the same sex is indicated (*p < 0.05), (**p < 0.01). Data represented as mean ± SEM.

MvD in leg skeletal muscle was assessed through exercise performance, photoacoustic imaging of tissue oxygen saturation (sO_2_), and blood flow in the femoral artery at rest (Figure 2). Treadmill performance revealed increased endurance in male control rats up to Month 5 and sustained endurance in female control rats up to Month 8, at which point the running distance began to decline with age. Furthermore, diabetic males and females displayed a consistently lower exercise tolerance relative to their control counterpart—significance between control and diabetic males was observed starting at Month 5, but significance between control and diabetic females was never attained throughout the 12 months. The lack of significance between diabetic and control females stems from the superior exercise capabilities of young, healthy female rats, where 6 of 8 control and 6 of 12 diabetic rats finished the 20-min regime at Month 4 post-induction without going into exhaustion, thus rendering it difficult to assess their true limit. In contrast, in the male cohort at Month 4 post-induction, only 1 of 8 control and 1 of 17 diabetic rats completed the 20-min regime.

Photoacoustic imaging revealed reduced skeletal muscle sO_2_ in diabetic males from as early as Month 4 post-induction, reaching significance at Month 10. Diabetic females also displayed a significant reduction in muscle sO_2_ at Month 10 but no changes prior. By Month 12, muscle sO_2_ declined in female controls as they advanced into middle age but, nonetheless, remained higher relative to diabetic females.

Baseline femoral artery blood flow measurement revealed a significant reduction in peak systolic blood velocity in diabetic males at Month 5 and diabetic females at Month 6, which was sustained to Month 12 for females. End-diastolic velocity was significantly decreased only in diabetic females at Month 12.

### Histopathology and Immunohistochemistry

**Figure 3:**
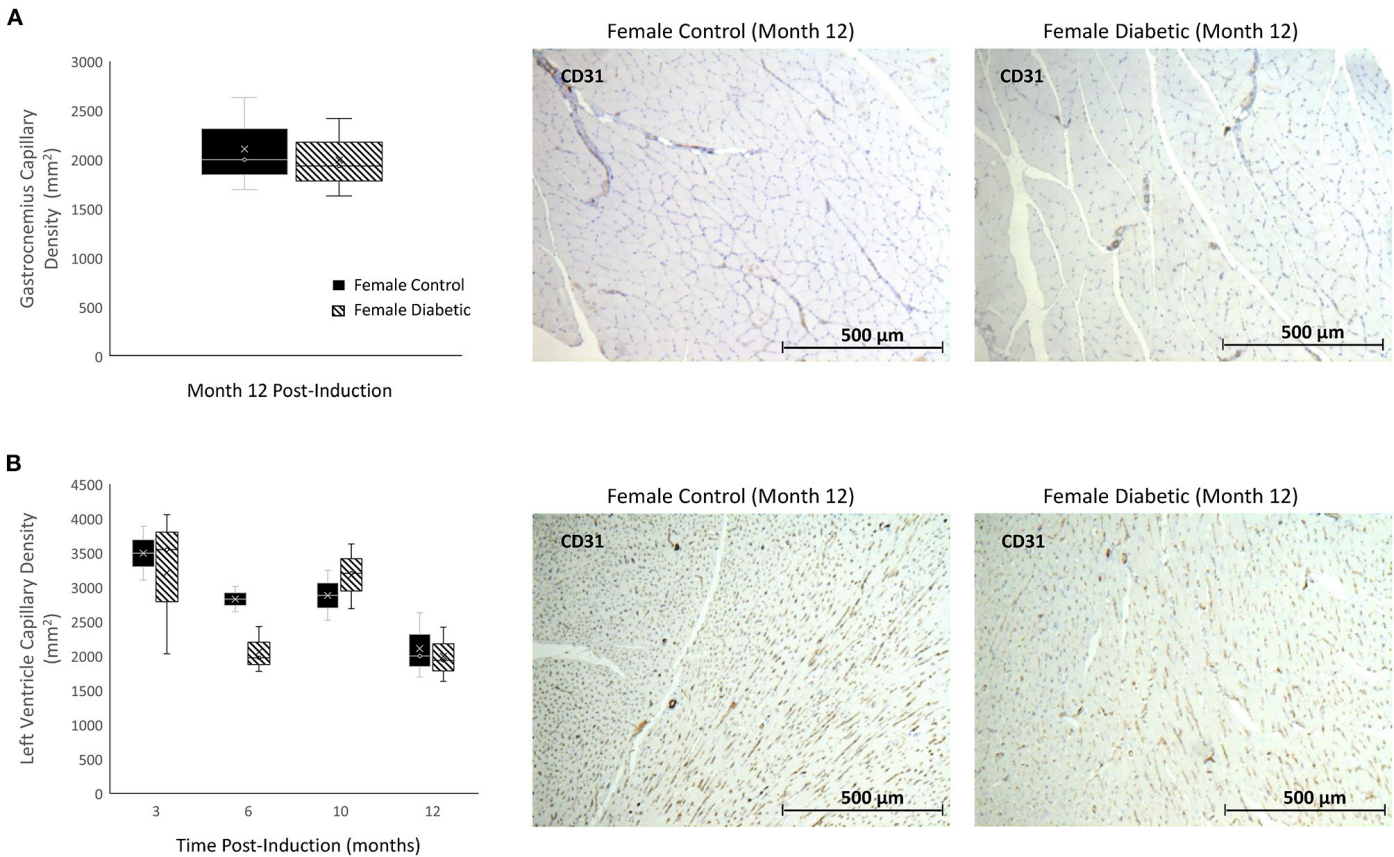
Histology of skeletal and myocardial muscle capillary density reveals no rarefaction. Timepoints refer to time post-induction at 3 months (*n* = 2 control female and *n* = 3 diabetic female), 6 months (*n* = 2 control female and *n* = 3 diabetic female), 10 months (*n* = 2 control female and *n* = 3 diabetic female), and 12 months (*n* = 3 control female and *n* = 3 diabetic female) post-induction. Gastrocnemius **(A)** and myocardial muscle **(B)** capillary density quantified as the number of microvessels per mm^2^ of tissue on CD31 stained sections. Representative images of skeletal and cardiac sections stained for CD31 from female control (middle) and diabetic (right) rats at 12 months post-induction. Boxplot mean value is represented with the symbol “X.” Results for male rats are shown in reference (8).

There were no signs of microvascular rarefaction in the heart or skeletal muscle, as measurement of capillary density showed no difference between female control and diabetic rats (Figure 3). Similarly, in males, differences in capillary density were absent in the heart and leg muscle (8).

**Figure 4:**
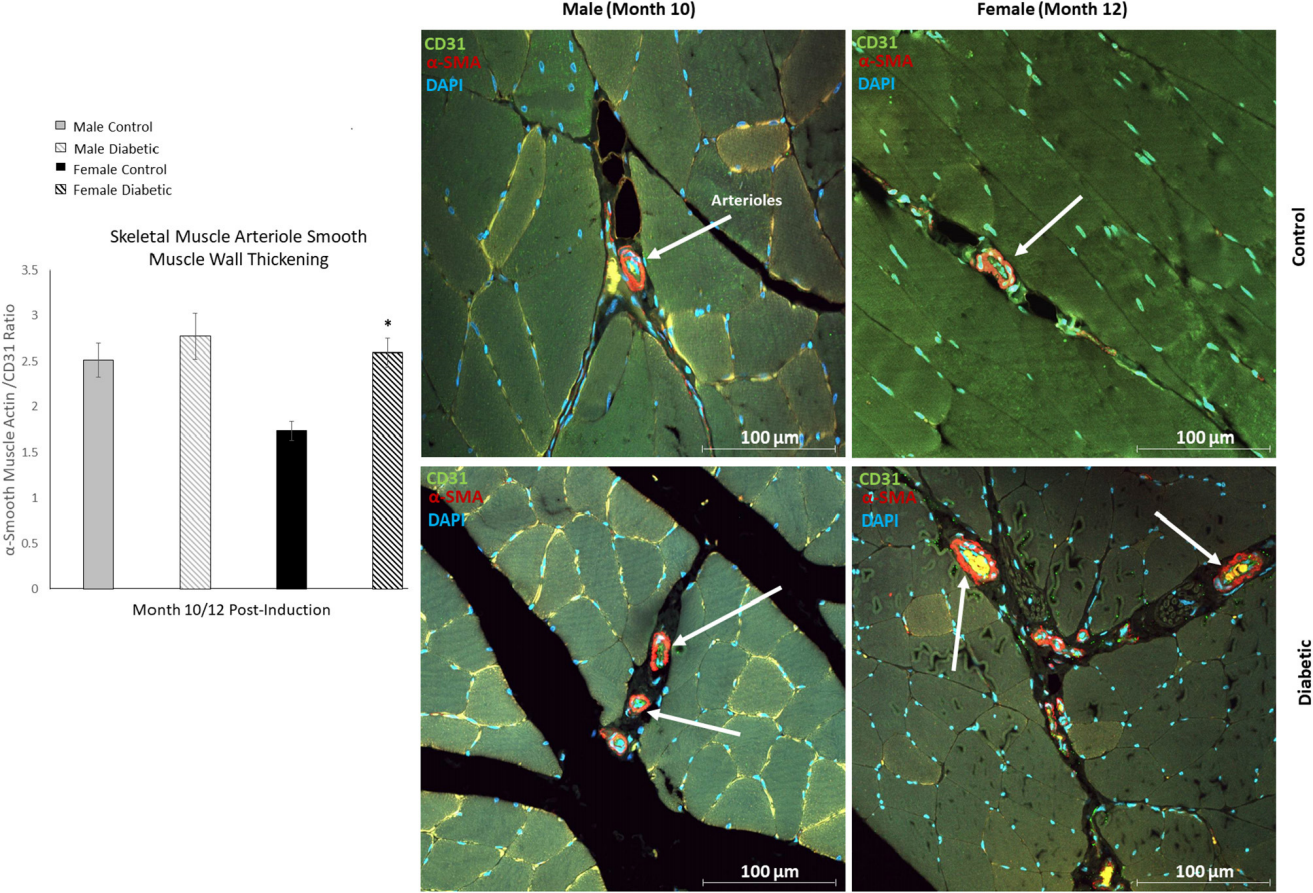
Immunofluorescence of small arteriole perivascular smooth muscle wall in leg skeletal muscle shows thickening in diabetic females. Quantification expressed as a ratio of surface area coverage of αSMA to CD31 for each arteriole (10–25 μm). Assessment conducted at 10 and 12 months post-induction for males and females, respectively (*n* = 3 control male, *n* = 4 diabetic male, *n* = 3 control female and *n* = 4 diabetic female). White arrows highlight representative arterioles from each cohort. Significance between control and diabetic cohorts with their respective sex is indicated (*p < 0.05). Data represented as mean ± SEM.

**Figure 5:**
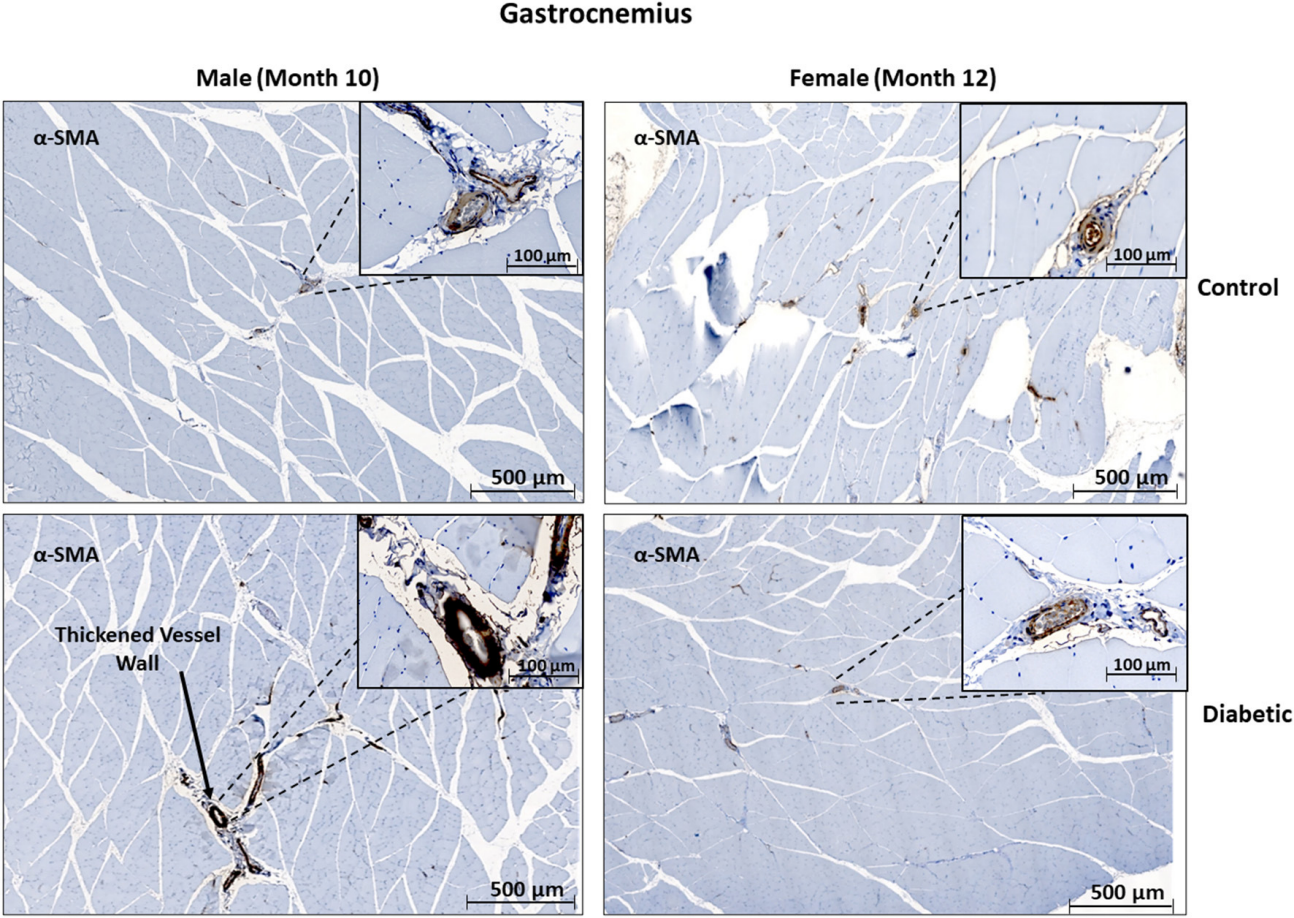
Histology of large arteriole perivascular smooth muscle wall in leg skeletal muscle shows thickening in diabetic males. Representative images of gastrocnemius skeletal muscle sections stained with αSMA in males at 10 months and females at 12 months post-induction. Images depict a thickened perivascular smooth muscle layer in the male diabetic cohort only.

In skeletal muscle, microangiopathy in small arterioles (10–25 μm in diameter) was assessed by evaluating the ratio of the surface areas of the perivascular smooth muscle layer to the endothelial lining. A significantly higher ratio of αSMA/CD31 emerged in the gastrocnemius in diabetic females, and only a slight but insignificant increase was observed in diabetic males (Figure 4). Absolute measurements of surface area confirmed that the increase in αSMA/CD31 ratios was attributed to an increase in smooth muscle wall thickness, with minimal changes in the endothelial lining. Macroangiopathy was observed in diabetic males only, where thickened vessel walls were visually noted in large arterioles and small arteries (25–100 μm in diameter) (Figure 5). This pathology was absent in the female cohort.

**Figure 6:**
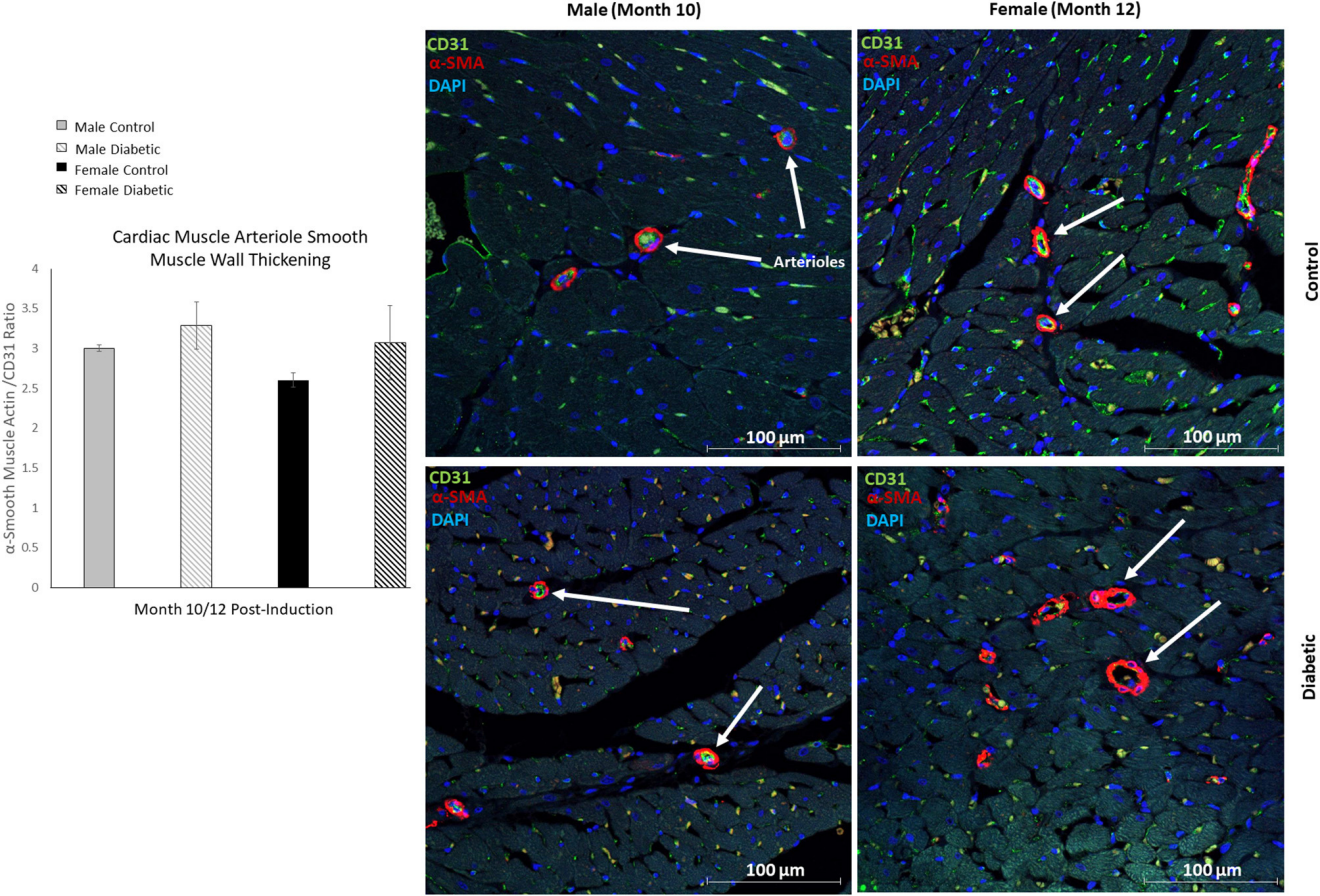
Immunofluorescence of small arteriole perivascular smooth muscle wall in the myocardium shows no thickening in either sex. Quantification expressed as a ratio of surface area coverage of αSMA to CD31 for each arteriole (10–25 μm). Assessment conducted at 10 and 12 months post-induction for males and females, respectively (*n* = 3 control male, *n* = 4 diabetic male, *n* = 3 control female and *n* = 4 diabetic female). White arrows highlight representative arterioles from each cohort. Data represented as mean ± SEM.

**Figure 7:**
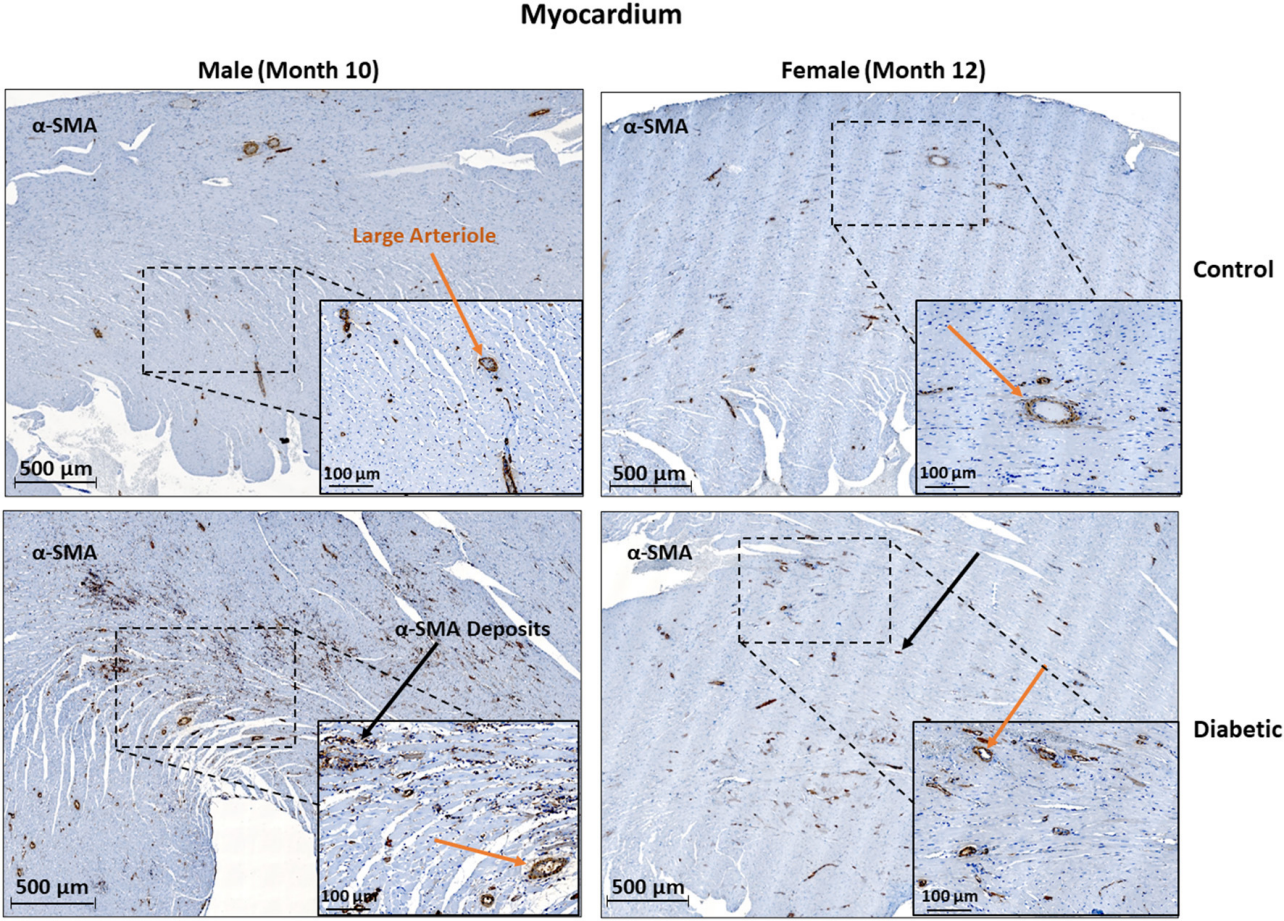
Histology of large arteriole perivascular smooth muscle wall in the myocardium shows no thickening in either sex. Representative images of LV cardiac muscle sections stained with αSMA in males at 10 months and females at 12 months post-induction. A substantial amount of myofibroblast deposits is seen in diabetic males and, to a lesser degree, in diabetic females (black arrows). No difference was observed in large arteriole smooth muscle wall thickness (orange arrows).

In cardiac muscle, similar assessment for microangiopathy revealed a non-significant increase in the αSMA/CD31 ratio in diabetic animals of both sexes (Figure 6). Macroangiopathy was also absent—in contrast to thicker smooth muscle coverage of large arterioles and small arteries in the leg muscle of diabetic males, a thickened smooth muscle layer was not observed in the myocardium of either sex (Figure 7). However, αSMA staining revealed prominent αSMA deposition in the LV of diabetic males and, to a lesser degree, diabetic females.

**Figure 8:**
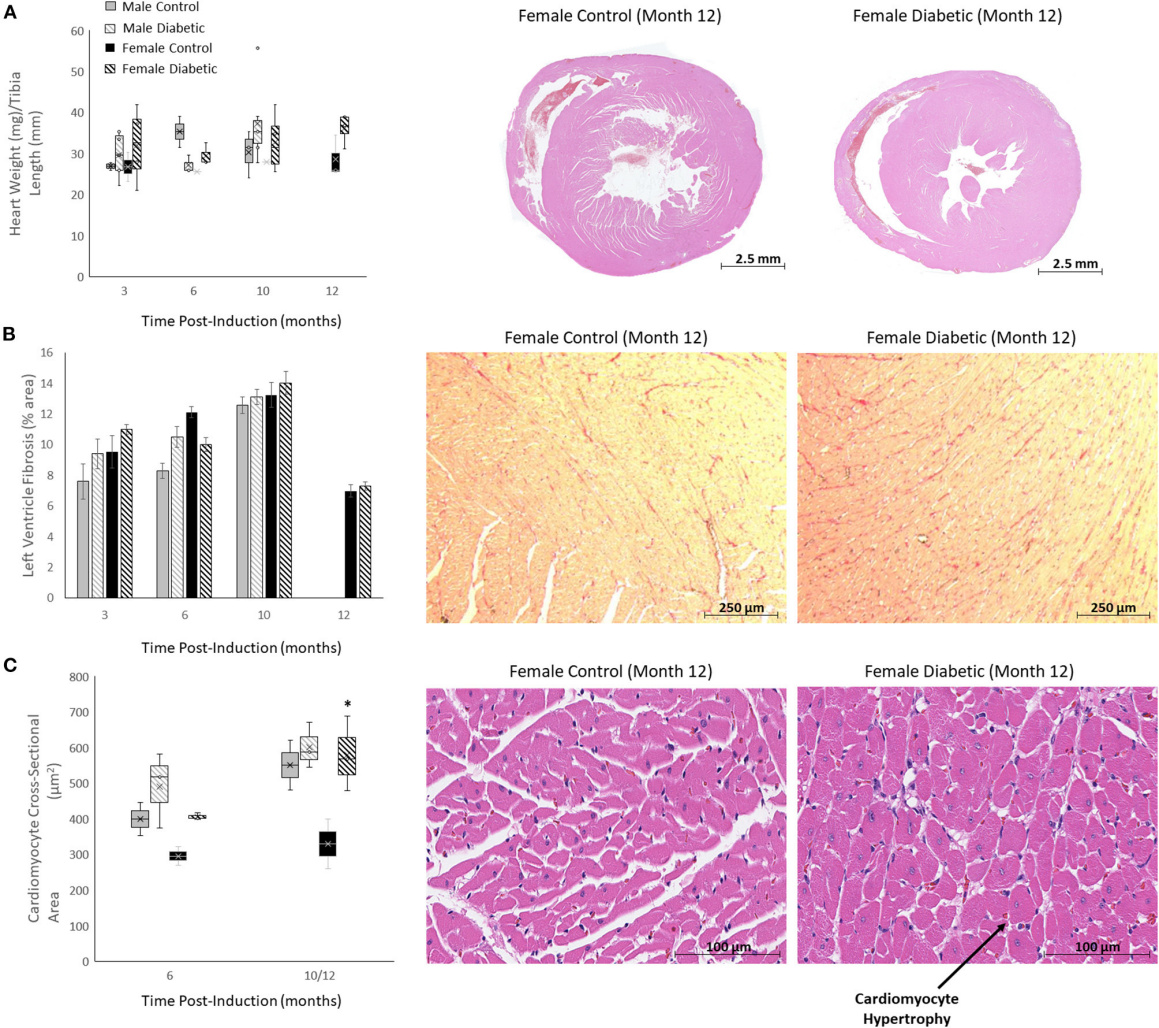
Histology of non-vascular markers in the heart. Timepoints refer to time post-induction at 3 months (*n* = 2 control male, *n* = 8 diabetic male, *n* = 2 control female, *n* = 3 diabetic female), 6 months (*n* = 2 control male, *n* = 3 diabetic male, *n* = 2 control female, *n* = 3 diabetic female), 10 months (*n* = 5 control male, *n* = 9 diabetic male, *n* = 2 control female, *n* = 3 diabetic female) and 12 months (*n* = 3 control female and *n* = 3 diabetic female). **(A)** Heart weight-to-tibia-length ratio (left), and H&E slide scans at 12 months post-induction of control and diabetic females (right). **(B)** Interstitial fibrosis quantified as percentage of collagen surface area coverage on stained heart sections (left), and representative images of control and diabetic female heart tissue stained for collagen at 12 months post-induction at 10x magnification (right). **(C)** Cardiomyocyte cross-sectional area quantified at 6 and 12 months post-induction (left), and representative images of control and diabetic female heart tissue stained with H&E at 12 months post-induction at 40x magnification (right). Histology images for male cohort is found in reference (8). Boxplot mean value is represented with the symbol “X.” Significance at individual timepoints between control and diabetic cohorts of the same sex is indicated (*p < 0.05). Data represented as mean ± SEM.

Histology for myocardial fibrosis and cardiomyocyte hypertrophy in the LV was performed throughout the study (Figure 8). Despite diabetic animals exhibiting a slightly elevated heart weight/tibia length ratio (Figure 8A), fibrosis was absent in diabetic animals of both sexes (Figure 8B). Cardiomyocyte hypertrophy was detected only in diabetic females at the late stage, with significance emerging relative to female control rats at Month 12 (Figure 8C). Refer to (8) for images acquired from male rats.

### Echocardiography

Echocardiography was performed monthly for 12 months post-induction in the female cohort (Supplementary Figures S1, S2), and parameters most commonly associated with systolic and diastolic dysfunction were compared against those in the male cohort from a previously published study (8) (Table 2). Echocardiographic data for the male cohort are in found in (8). An evolving E/A ratio, which helps demarcate four grades of diastolic dysfunction, did not manifest in the female diabetes cohort as it did in the male diabetes cohort. Instead, we observed a decrease in the E wave from Month 3–6, consistent with grade 1 diastolic dysfunction (15). An increase in IVRT and decrease in the pulmonary diastolic venous wave, both features of diastolic dysfunction, appeared in both male and female diabetic animals at similar time intervals. Mild systolic dysfunction, evaluated from an increased IVCT, increased ejection time, and decreased pulmonary systolic venous wave, was confirmed in both sexes. A preserved LV ejection fraction was maintained in both sexes throughout the study, although a slightly reduced ejection fraction was noted in both diabetic cohorts. Other structural parameters, including a decreased fractional shortening, enlarged LV, and increased LV mass index, presented in both diabetic cohorts at similar time intervals. The relative LV wall thickness was reduced in the male diabetic group only. Strain analysis conducted up to Month 12 in both sexes showed no difference between control and diabetic groups (Supplementary Figure S3).

**Table 2:** Cardiac function and morphology on echocardiography in diabetic rats.

**Pulsed Wave Doppler**	**Male**	**Female**	**M-Mode Echocardiography**	**Male**	**Female**
MV E/A Increase/decrease	↓ (month 4) ↑ (month 8)	↓ in E only	Ejection Fraction ≥ 50%	Yes	Yes
IVRT Increase	Yes	Yes	Fractional Shortening Decrease	Yes	Yes
Pulmonary Diastolic Wave Decrease	Yes	Yes	LV Systolic Diameter Increase	Yes	Yes
IVCT Increase	Yes	Yes	LV Diastolic Diameter Increase	Yes	Yes
Ejection Time Increase	Yes	Yes	Relative Wall Thickness Decrease	Yes	No
Pulmonary Systolic
Wave Decrease	Yes	Yes	LV Mass Index Increase	Yes	Yes

*Parameters measured from pulsed wave Doppler include: mitral valve E/A ratio representing the ratio between peak blood velocity in the left ventricle (LV) during early diastole to peak blood velocity in the LV at late diastole, isovolumetric relaxation time (IVRT), pulmonary peak diastolic (D) venous wave, isovolumetric contraction time (IVCT), LV ejection time, and pulmonary peak systolic (S) venous wave. Parameters measured from M-mode echocardiography include, LV ejection fraction, fractional shortening, LV diameter at end-systole, LV diameter at end-diastole, relative wall thickness, and LV mass index*.

### Confirmation of Diabetes and Blood Chemistry

A sustained level of hyperglycemia was confirmed in the female diabetic cohort, with blood glucose significantly elevated from week 5 to 11 months post-induction. Further confirmation of type II diabetes was established through glucose tolerance and insulin resistance testing (Supplementary Figure S4). It is important to note that obesity was not a phenotype in our model, as diabetic and control animals gained weight at a similar rate (Supplementary Figure S4). The development of cataracts in female diabetic rats was noted as early as 2 to 3 months post-induction, similar to observations in males (8). Cardiac blood panel analysis revealed no increase in total cholesterol in female diabetics, in to contrast to diabetic males, and no increase in triglycerides throughout the study (Supplementary Figure S5). Kidney blood panel analysis used clinically to diagnose kidney damage (19) showed no difference in albumin or total plasma protein levels between control and diabetic rats in either sex (Supplementary Figure S5).

### Femoral Artery Flow-Mediated Dilation

Femoral artery FMD measurements at Month 5, 6, and 12 revealed a significant increase in blood flow upon cuff release in both the control and diabetic cohort, but the percentage increase was not different between the two cohorts or sexes (Supplementary Figure S6). This observation confirms that while baseline blood flow in the femoral artery was significantly reduced in diabetic animals (see section 3.1), femoral artery vasoreactivity was unaffected. Interestingly, arterial oxygen saturation measured in female rats at Month 12 using a rat foot sensor revealed a notable decrease in the diabetic cohort relative to control (81.0% ± 8.9% vs. 93.4% ± 4.7%).

### Cardiac Perfusion

Cardiac perfusion assessment on photoacoustic imaging revealed no difference between control and diabetic cohorts in either sex (Supplementary Figure S7). Myocardial sO_2_ levels were consistent between female control/diabetics throughout the study. Male diabetic rats had a slightly higher but non-significant myocardial sO_2_ compared to controls.

### Sex Differences in Key Biomarkers

A sex comparison revealed that except for a handful of measured parameters, the majority did not exhibit sex-related differences. A key exception was in treadmill tolerance, where females ran significantly longer than males in the respective control and diabetic groups (Table 3). Another exception was in leg photoacoustic measurements: resting sO_2_ was significantly higher in male controls relative to females, but the difference was abolished when comparing male to female diabetic animals. In the myocardium, resting sO_2_ was significantly higher in females compared to males. Echocardiographic parameters displayed no sex differences. Finally, from blood chemistry, HDL, LDL, and total cholesterol showed a sex-related difference, with diabetic males consistently displaying significantly higher levels than diabetic females.

**Table 3:** Key parameters exhibiting sex-dependent differences.

**Parameter**	**Description of difference**
Treadmill running distance	Females run longer or without going into exhaustion.
Leg muscle arteriole smooth muscle thickening	Small arteriole thickening in females, large arteriole thickening in males.
Leg photoacoustic sO_2_	Control male rats have higher sO_2_ than females.
Heart photoacoustic sO_2_	Females have higher sO_2_ than males.
Hyperlipidemia	HDL, LDL, and total cholesterol escalate in diabetic males, but no changes are seen in diabetic females.
Kidney function	Creatine levels decline in diabetic females; insignificant elevation in diabetic males.

**Figure 9:**
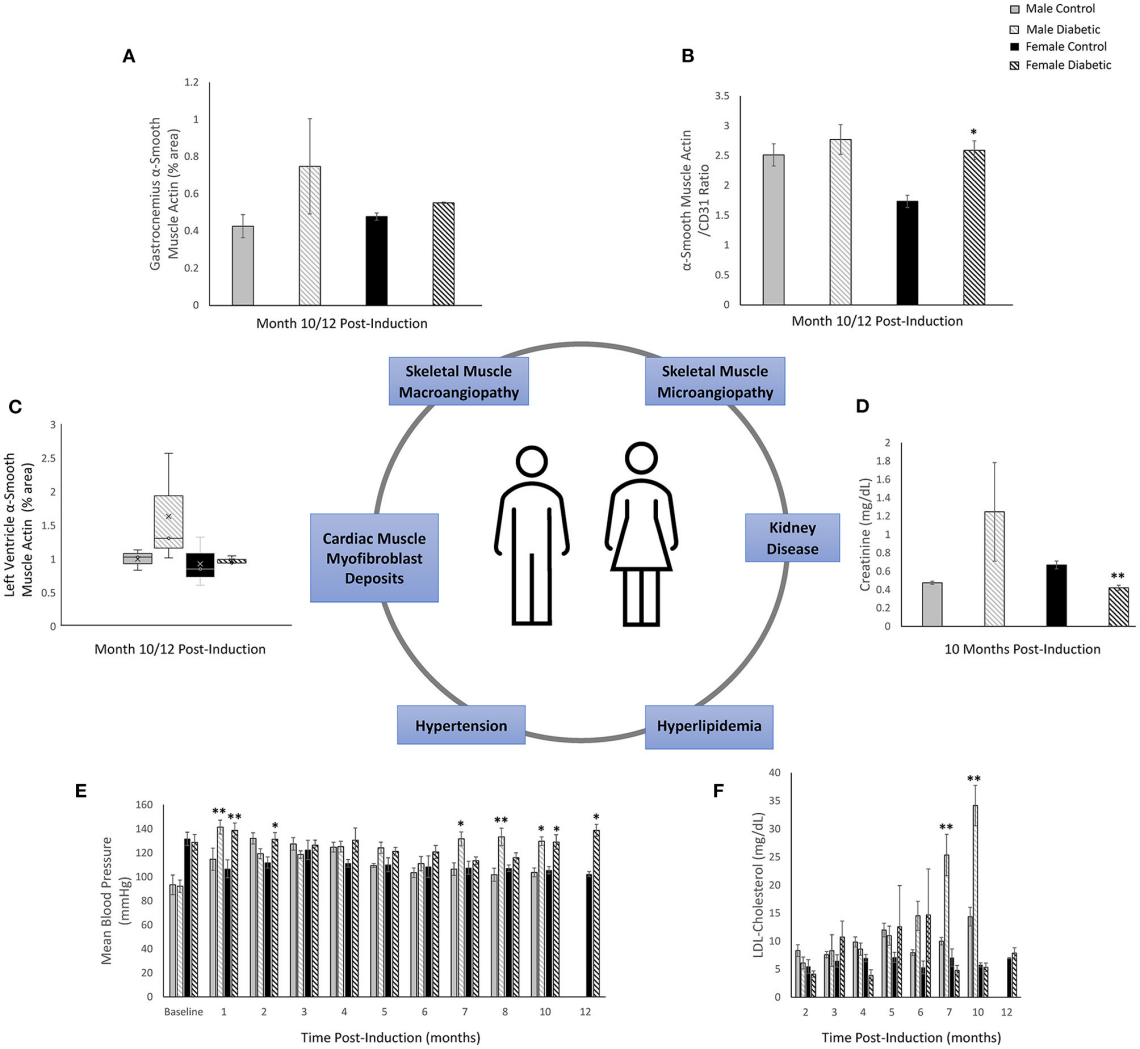
Sex-related differences in key biomarkers in diabetic cardiomyopathy. **(A)** Leg skeletal muscle (gastrocnemius) large arteriole wall thickness quantified as percentage of αSMA surface area coverage at Month 10 (*n* = 3 control male and *n* = 3 diabetic male) and Month 12 (*n* = 3 control female and *n* = 3 diabetic female) post-induction. **(B)** Leg skeletal muscle small arteriole smooth muscle wall thickening, quantified as a ratio of surface area coverage of αSMA to CD31. Assessment conducted at Month 10 (*n* = 3 control male and *n* = 4 diabetic male) and Month 12 (*n* = 3 control female and *n* = 4 diabetic female) post-induction. **(C)** LV myofibroblast deposits quantified as percentage of αSMA surface area coverage on cardiac muscle sections at Month 10 (*n* = 3 control male and *n* = 3 diabetic male) and Month 12 (*n* = 3 control female and *n* = 3 diabetic female) post-induction. **(D)** Kidney disease evaluated through blood plasma creatinine excretion levels at Month 10 (*n* = 4 control male, *n* = 5 diabetic male, *n* = 4 control female, and *n* = 5 diabetic female) post-induction. **(E)** Hypertension measured though non-invasive tail cuff to determine mean blood pressure in all rats. **(F)** Hyperlipidemia quantified from low-density lipoprotein (LDL) cholesterol over time. Data was obtained at Month 2 (*n* = 3 each of control male, diabetic male, control female, and diabetic female), Month 3–7 (*n* = 5 each of control male, diabetic male, control female, and diabetic female), Month 10 (*n* = 4 control male, *n* = 6 diabetic male, *n* = 4 control female, and *n* = 5 diabetic female), and Month 12 (*n* = 3 control female and *n* = 5 diabetic female) post-induction. Boxplot mean value is represented with the symbol “X.” Significance at individual timepoints between control and diabetic cohorts of the same sex is indicated (*p < 0.05), (**p < 0.01). Data represented as mean ± SEM.

Figure 9 summarizes diagrammatically the main findings on microvascular dysfunction and several biomarkers used in clinical workup (e.g., hypertension, hyperlipidemia). Sex-related differences are noted where applicable. Microvascular dysfunction underlying decreased leg skeletal muscle perfusion in diabetic males manifested as perivascular smooth muscle thickening in larger blood vessels (25–100 μm) only (Figure 9A). In diabetic females, microvascular dysfunction manifested as perivascular smooth muscle thickening in small arterioles (10–25 μm) only (Figure 9B). Perivascular smooth muscle thickening was not observed in the heart of either sex (see Section 3.2). However, staining for αSMA in the myocardium did reveal myofibroblast deposits in the LV of diabetic animals, with a higher αSMA content measured in diabetic males compared to females (Figure 9C). Chronic kidney disease evaluated through blood plasma creatinine (16) showed a non-significant elevation in male diabetic rats only. Diabetic females had significantly lower creatinine levels, an observation that has been documented previously in diabetic patients with kidney failure and poor muscle quality (17) (Figure 9D). Hypertension and hyperlipidemia, both routine biomarkers for evaluating risks for heart disease (18), presented late-stage in our diabetic cardiomyopathy model. Hypertension did not set in until Month 7 and 9 post-induction for diabetic male and female rats, respectively (Figure 9E). The transient spikes measured in the early timepoints are most likely attributed to stress from multiple STZ injections and from fasting/blood collection procedures. Significantly elevated LDL-cholesterol was observed only in diabetic males starting at Month 7; diabetic females showed no signs of hyperlipidemia at all (Figure 9F).

**Figure 10:**
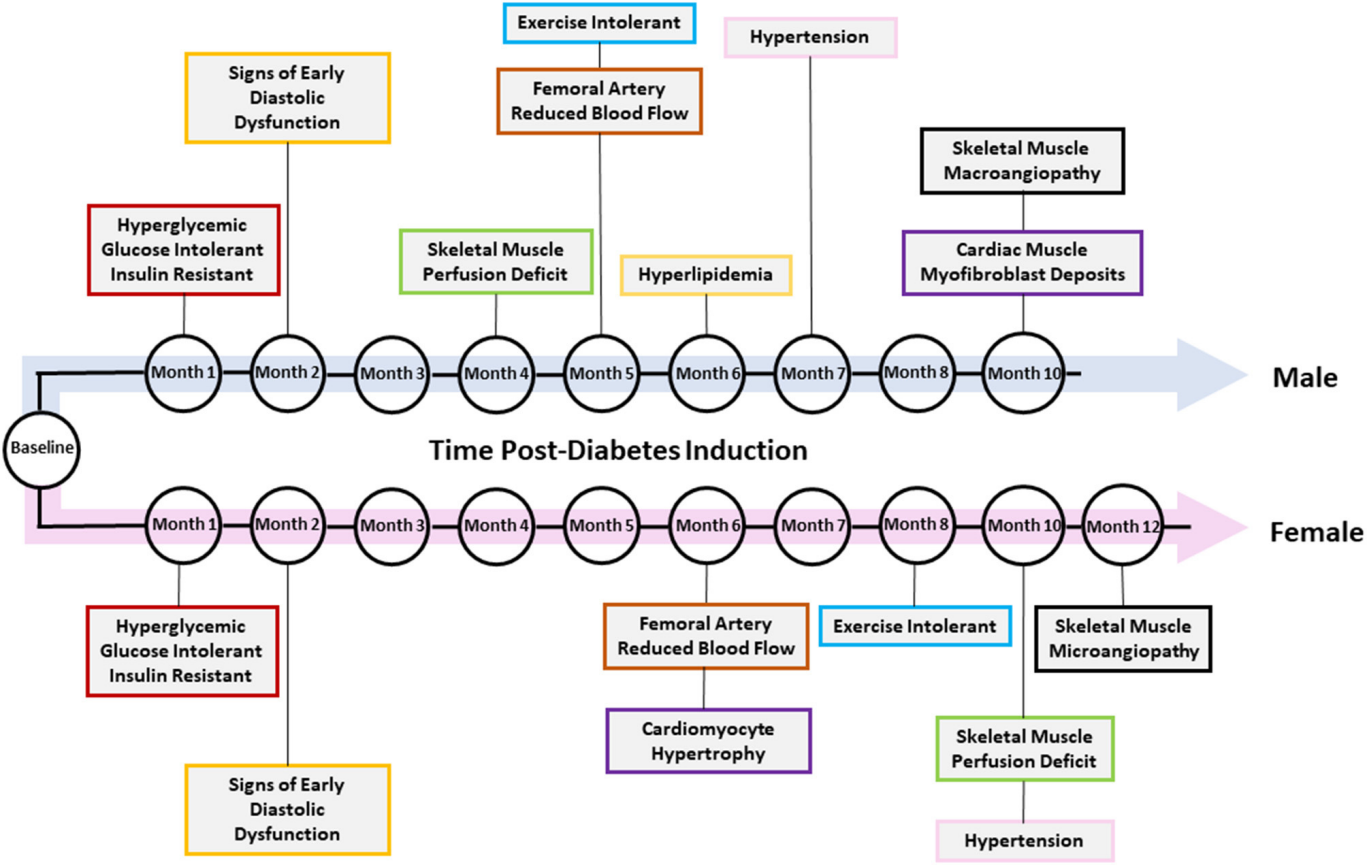
Temporal presentation of signs and symptoms of diabetic cardiomyopathy. The key changes in vascular function, exercise tolerance, cardiac function, and other heart failure-related biomarkers are shown for diabetic male and female rats. Note that not all changes were present in both sexes.

Figure 10 summarizes the temporal presentation of symptoms for both sexes for the 1-year study.

## Discussion

Diabetes is a significant risk factor for developing heart failure of both the HFpEF and HFrEF phenotype. While a key distinction between HFpEF and HFrEF is the preservation of a normal LV ejection fraction or lack thereof, they also share many similarities, such as exercise intolerance (20), imbalance of vasoactive hormones, metabolic deficiencies in vascular smooth muscle, and endothelial impairment (21). In our recently published study, we uncovered exercise intolerance as an early indicator of skeletal muscle mvD in male rats with diabetic cardiomyopathy and a preserved ejection fraction (8). This finding was supported by a reduced tissue oxygen saturation in skeletal muscle and a reduced femoral arterial blood flow. Most importantly, longitudinal monitoring revealed that skeletal muscle mvD manifested prior to the onset of heart failure symptoms or clinically assessed biomarkers, such as hypertension or elevated cholesterol. In the current study, we perform a deeper dive into the morphological changes in leg skeletal and cardiac muscle vasculature. We also include a side-by-side sex comparison of disease progression to determine if females are protected from microvascular complications (22).

Exercise intolerance was the earliest symptom to present in diabetic male rats but not females. Treadmill performance over 1 year revealed that diabetic males exhibited diminished exercise capacity as early as 4 months post-diabetes induction and became progressively more intolerant with time. On the other hand, female diabetic rats displayed evidence of lower exercise tolerance at Month 8, but the difference was not significant. We also noted that female rats, on the whole, had significantly better tolerance than males, with female control rats running the entire 20-min interval without exhaustion and significantly outperforming male controls. However, as both female and male controls became older, their exercise capacity declined, eventually matching that of the respective diabetic cohort.

Photoacoustic imaging of skeletal muscle tissue sO_2_ was able to reveal a difference between control and diabetic animals at Month 4 post-induction for males but not until Month 10 for females. It is important to note that we used photoacoustic imaging as a surrogate for perfusion; the indirectness of this measurement very likely underlies our inability to detect impaired leg perfusion in diabetic females at early times. While it is reasonable to assume a higher sO_2_ is a result of greater flow of blood into a region of tissue, oxygen saturation can be affected by factors independent of perfusion—for instance, oxygen metabolism. It is quite probable that severe microvascular complications need to set in to meet the sensitivity threshold of photoacoustic imaging. There is also the possibility estrogen protected the microvasculature, and it was not until after menopause, which is around Month 10 for rats (23, 24), when photoacoustic detected the reduced muscle perfusion seen here and in another study (25).

Despite the late presentation of photoacoustic changes in diabetic females, femoral arterial blood flow was reduced in diabetic rats of both sexes circa Month 5/6 post-induction. It is noteworthy that this timepoint coincides, at least in males, with the first emergence of a significant difference in exercise tolerance between control and diabetic animals. It is possible that exercise tolerance was also reduced significantly in female diabetic rats at Month 5/6, but significance relative to control females could not be obtained due to the natural ability of females to run the full routine without exhaustion. Our findings are very similar to a clinical study in type II diabetes patients in whom femoral arterial blood flow was assessed on magnetic resonance imaging (MRI) during low-intensity exercise (26). The authors concluded that femoral arterial flow was reduced despite the patients' maintaining a preserved cardiac output, suggesting impaired vascular function was independent of cardiac performance and reduced blood flow could result from impaired microvascular vasoreactivity.

Histopathology confirmed microvascular abnormalities underlying reduced leg skeletal muscle perfusion. First and foremost, microvascular rarefaction was absent in skeletal and cardiac muscle—this is not inconsistent with rarefaction reported in the literature, where assessments are typically made in genetic models in which late and severe symptoms of heart failure manifest within a short interval (27). In the current study, substantial perivascular smooth muscle thickening was observed around arterioles in diabetic animals. In diabetic female rats, a thickened smooth muscle layer was found around small arterioles, whereas a thickened smooth muscle layer was found around bigger arterioles in diabetic males. This sex-related finding in how microvascular dysfunction manifests differently was expected, especially in light of differences in heart failure progression reported in males and females (27). Diabetic women do have worse microvascular outcome and a higher mortality rate compared to men, particularly after menopause (22, 28). Furthermore, clinical studies have shown that diabetic men are more likely than women to present with macrovascular complications in their mid-60 s (10).

Microvascular histopathology in the heart revealed distinct differences from that in skeletal muscle. Perivascular smooth muscle thickening around small arterioles in diabetic animals was minimal. Unlike leg skeletal muscle microvasculature, the thickening in the heart was insignificant albeit perceptible, especially in diabetic female hearts. There was also no smooth muscle thickening around larger arterioles and small arteries in either sex. Taken together, these results suggest that leg skeletal muscle vasculature is more susceptible to early abnormal changes compared to myocardial vasculature, and that the heart vascular bed is one of the later ones to become diseased. Had a monitoring interval longer than a year been possible, significant perivascular muscle thickening and even rarefaction in the myocardium may have eventually presented.

Interestingly, a large amount of αSMA deposits from myofibroblasts was present at Month 10 post-induction in diabetic males and, to a lesser extent, in diabetic females. This finding is important, because collagen staining had revealed minimal interstitial fibrosis in the diabetic heart of both sexes, whereas αSMA staining confirmed cardiac injury (29). From this observation, we can deduce that interstitial fibrosis is also a later-stage marker of a diseased myocardium. Another notable observation was cardiomyocyte hypertrophy: this phenotype was not observed in diabetic males but observed strictly in diabetic females as early as 6 months post-induction and prior to the onset of hypertension.

Comparing our new mvD biomarker against clinical markers used routinely yields several interesting findings. Cardiac blood markers (e.g., LDL-cholesterol, total cholesterol, triglycerides) are early indicators of risk for heart failure; however, these were elevated in diabetic males only during late stage, and diabetic females had normal blood chemistry throughout. Hypertension, another early indicator of risk, presented actually very late in both sexes, with diabetic females developing hypertension only upon menopause. Echocardiography revealed signs of heart failure with a preserved ejection fraction, systolic and diastolic dysfunction, and a dilated LV, with similar temporal profiles in both sexes. No change was detected on strain imaging. It is important to note that because hypertension developed naturally in our non-genetic model and appeared late, recapitulating all the signs of a clinical HFpEF diagnosis (e.g., thickened LV wall) may require a much longer study than is possible in rats. What is clear from our model is that diabetic cardiomyopathy is a slow process, with exercise intolerance appearing early due to microvascular smooth muscle thickening in skeletal muscle, slow onset of hypertension and hyperlipidemia, insignificant microvascular changes in the heart coincident with late hypertension, and cardiac changes consistent with a preserved ejection fraction but lacking some features of a fully HFpEF heart. Our model recapitulates the earliest events that take place in the progression of diabetic cardiomyopathy, therefore providing a time-resolved dataset of pathological changes in different tissues.

On blood test of renal function, diabetic males had a non-significant increase in creatinine levels at Month 10. In contrast, diabetic females had an even lower creatinine level compared to control females at Month 10. This finding in females agrees with clinical studies demonstrating that diabetic patients on hemodialysis have low serum creatinine due to reduced muscle strength and mass (17). Given that skeletal muscle is responsible for 75% of insulin-mediated glucose disposal, the presence of chronic insulin resistance will severely impact muscle mass, and thus reducing creatine excretion and increasing the probability of major adverse cardiac events (30).

Several limitations were present in this study and require mention. Terminal pressure-volume experiments were not conducted in the heart to measure LV pressure overload. Echocardiography was performed on anesthetized rats with no stress testing incorporated. Cardiac blood panels did not include any markers for inflammation or brain type natriuretic peptide (BNP), although new studies are suggesting that BNP should not be considered a gold standard marker for diagnosing HFpEF (31, 32). The number of animals for histological analysis could be higher; however, with ANOVA analysis, reporting of significance is conservative, and one can be confident of any metric reported to be different. The study timeline also could not be extended to examine the longer-term impact of hypertension on the heart and vasculature due to considerations for the welfare of animals who were severely diabetic. Had it been possible to extend the study duration, the classical signs of HFpEF (e.g., concentric hypertrophy, fibrosis, microvascular rarefaction) may have emerged. Finally, we made the curious observation of a declining non-fasted blood glucose toward the end of the one-year study in both male and female diabetic animals. This was an unexpected finding, but weight loss was ruled out as a culprit, as animals maintained their weight. Interestingly, a similar observation was also reported in a similar type II diabetes model (33), where the authors confirmed blood glucose recovery was not a result of increased insulin production. Further investigation is required to understand the importance of this observation.

In conclusion, we have uncovered sex-dependent, vascular morphological changes underlying microvascular dysfunction in a diabetic cardiomyopathy model in rats with multiple overlaps with the HFpEF phenotype. Exercise intolerance is the earliest symptom to emerge in diabetic males, later in females, both manifesting at a time when echocardiography indicates a preserved ejection fraction, early diastolic dysfunction, and mild systolic dysfunction. Histopathology revealed abnormal thickening of the perivascular smooth muscle layer in leg skeletal muscle around small arterioles (10–25 μm) in diabetic females and around large arterioles (25–100 μm) in diabetic males. Significant smooth muscle thickening in the myocardial vasculature was absent. Vessel rarefaction was also absent in both the heart and leg skeletal muscle. Clinically used femoral arterial FMD showed that while the reactivity was unaffected in diabetic subjects, both diabetic males and females showed a lower baseline blood flow in the leg as early as Month 5/6. Photoacoustic imaging of tissue sO_2_ was sensitive to lower leg perfusion at Month 4 in diabetic males but at Month 10 in diabetic females. At the final timepoint of the study (Month 10 for males and Month 12 for females), clinically used biomarkers such as hypertension and hyperlipidemia either had just appeared or not at all. Commonly cited signs of HFpEF (e.g., myocardial fibrosis, rarefaction, LV wall thickening) had not developed by the final timepoint.

## Data Availability Statement

The original contributions presented in the study are included in the article/Supplementary Material, further inquiries can be directed to the corresponding author.

## Ethics Statement

The animal study was reviewed and approved by our institutional Animal Care Committee (Division of Comparative Medicine) at University of Toronto (protocol #20012191), and all procedures were conducted in accordance with the Canadian Council on Animal Care.

## Author Contributions

SL contributed to the study design, performing all animal procedures and imaging, data analysis, statistical analysis, and drafting and editing of the manuscript. XS contributed to immunohistochemistry and immunofluorescence protocol development and tissue staining. MH, ML, and HY all contributed to expertise on histological preparation and analysis. SN contributed to direction of immunohistochemistry and immunofluorescence. H-LC contributed to the overall direction, conceptualization, funding, study design, statistical analysis, and editing of the manuscript. All authors read and approved the final manuscript.

## Funding

This work was supported by the Canadian Institutes of Health Research [Grant Number #PJT-175131 to H-LC]; Natural Sciences and Engineering Research Council of Canada [Grant Number #RGPIN-2019-06137 to H-LC]. Canada Foundation for Innovation/Ontario Research Fund [Grant #34038 to H-LC]. Dean's Spark Professorship [to H-LC]; Ontario Graduate Scholarship [to SL].

## Conflict of Interest

The authors declare that the research was conducted in the absence of any commercial or financial relationships that could be construed as a potential conflict of interest.

## Publisher's Note

All claims expressed in this article are solely those of the authors and do not necessarily represent those of their affiliated organizations, or those of the publisher, the editors and the reviewers. Any product that may be evaluated in this article, or claim that may be made by its manufacturer, is not guaranteed or endorsed by the publisher.

## Supplementary Material

Supplementary Figure S1 Female cardiac function and LV dimensions measured on pulsed wave Doppler and M-mode echocardiography**. (A)** Mitral valve E/A ratio representing the ratio between peak blood velocity in the left ventricle (LV) during early diastole to peak blood velocity in the LV at late diastole. **(B)** Isovolumetric relaxation time (IVRT). **(C)** Isovolumetric contraction time (IVCT). **(D)** LV ejection time. **(E)** LV myocardial performance index (MPI). **(F)** Pulmonary peak diastolic (D) venous wave. **(G)** Pulmonary peak systolic (S) venous wave. **(H)** Left ventricle (LV) ejection fraction. **(I)** Fractional shortening. **(J)** LV diameter at end-systole. **(K)** LV diameter at end-diastole. **(L)** Relative wall thickness (RWT). **(M)** LV mass index. Corresponding data for male cohort is found in reference (8). Significance at individual timepoints is indicated (^*^*p* < 0.05), (^**^*p* < 0.01). Data represented as mean ± SEM.

Supplementary Figure S2 Additional echocardiographic parameters for female rats. (**A)** LV stroke volume calculated as EDV-ESV. **(B)** LV cardiac output calculated as heart rate × stroke volume. **(C)** Mitral valve peak blood velocity in the LV during early diastole (E) wave **(D)** Mitral valve peak blood velocity in the LV during late diastole (A) wave **(E)** Mitral valve E/e' ratio representing atrial filling pressure, defined as the ratio between peak blood velocity in the LV and peak mitral annular velocity during early diastole. **(F)** LV anterior wall thickness at end-systole. **(G)** LV anterior wall thickness at end-diastole **(H)** LV posterior wall thickness at end-systole. **(I)** LV posterior wall thickness at end-diastole. **(J)** LV volume at end-systole. **(K)** LV volume at end-diastole. **(L)** LV Filling time. **(M)** LV mass. **(N)** Unconscious heart rate (beats per minute [bpm]). Corresponding data for male cohort is found in reference (8). Significance at individual timepoints is indicated (^*^*p* < 0.05), (^**^*p* < 0.01). Data represented as mean ± SEM.

Supplementary Figure S3 Strain analysis on echocardiography. Global and endocardium longitudinal strain, and radial strain were analyzed from the LV at Month 6 (*n* = 5 control male, *n* = 7 diabetic male, *n* = 2 control female, and *n* = 4 diabetic female), Month 10 (*n* = 3 control male, *n* = 7 diabetic male, *n* = 3 control female, and *n* = 3 diabetic female), and Month 12 (*n* = 3 control female and *n* = 5 diabetic female) post-induction. Data represented as mean ± SEM.

Supplementary Figure S4 Body weight, blood glucose, confirmation of type II diabetes, and non-invasive blood pressure measurements. **(A)** Weekly weight measurements in all control and diabetic female rats. **(B)** Weekly non-fasting blood glucose measurements in all control and diabetic female rats. **(C)** Oral glucose tolerance test performed after overnight fasting in female control (*n* = 5) and diabetic (*n* = 5) rats immediately following the diabetes induction period. **(D)** Insulin tolerance testing performed after 6-h fasting in female control (*n* = 5) and diabetic (*n* =5) rats one week post-induction. **(E)** Blood insulin levels measured from plasma collected after overnight fasting (μIU/mL) and Homeostatic Model Assessment of Insulin Resistance (HOMA-IR) calculated for female control (*n* = 4) and diabetic (*n* = 3) rats. **(F)** Systolic blood pressure over time. **(G)** Diastolic blood pressure over time. Corresponding data for male cohort is found in reference (8). Significance at individual timepoints between control and diabetic cohorts of the same sex is indicated (^*^*p* < 0.05), (^**^*p* < 0.01). Data represented as mean ± SEM.

Supplementary Figure S5 Cardiac and kidney blood panels. **(A)** High-density lipoprotein (HDL) cholesterol over time (top left). Total cholesterol over time (top right). Triglyceride levels over time (bottom left). Fasting glucose blood levels over time (bottom right). Data was obtained from Month 2 (*n* = 3 each of control male, diabetic male, control female, and diabetic female), Moth 3–7 (*n* = 5 each of control male, diabetic male, control female, and diabetic female), Month 10 (*n* = 4 control male, *n* = 6 diabetic male, *n* = 4 control female, and *n* = 5 diabetic female), and Month 12 (*n* = 3 control female and *n* = 5 diabetic female) post-induction. **(B)** Albumin levels (top) and total protein levels (bottom) at Month 10 (*n* = 4 control male, *n* = 5 diabetic male, *n* = 4 control female, and *n* = 5 diabetic female) post-induction. Significance between control and diabetic cohorts of the same sex at individual timepoints is indicated (^*^*p* < 0.05), (^**^*p* < 0.01). Data represented as mean ± SEM.

Supplementary Figure S6 Femoral arterial flow-mediated dilation on Doppler ultrasound. Peak systolic velocity (left) and end-diastolic velocity (right) at Month 6 (*n* = 5 each of control male, diabetic male, control female, and diabetic female) post-induction in male (top) and female (bottom) cohorts. Significance was evaluated between pre-cuffing (5 min) and cuff release (6 min), and difference was observed for both control and diabetic cohorts. Significance is indicated (^*^*p* < 0.05). Data represented as mean ± SEM.

Supplementary Figure S7 Photoacoustic imaging of myocardial oxygen saturation shows no change in either sex. Tissue oxygen saturation in the myocardium at Month 8 (*n* = 3 control male, *n* = 5 diabetic male, *n* = 5 control female, *n* = 5 diabetic female), Month 10 (*n* = 4 control male, *n* = 6 diabetic male, *n* = 5 control female, and *n* = 6 diabetic female), and Month 12 (*n* = 3 control female and *n* = 5 diabetic female) post-induction. Photoacoustic ultrasound images of myocardium in male and female control (top) and diabetic (bottom) rats at Month 10 and 12 post-induction, respectively. Greyscale images are B-Mode images; colormaps are photoacoustic tissue oxygenation saturation overlay.
