# Supplementary figures and images for: Microvascular Dysfunction in Skeletal Muscle Precedes Myocardial Vascular Changes in Diabetic Cardiomyopathy: Sex-Dependent Differences
Source: Front Cardiovasc Med. 2022 May 18;9:886687. doi: 10.3389/fcvm.2022.886687 (PMC9157579; doi:10.3389/fcvm.2022.886687)

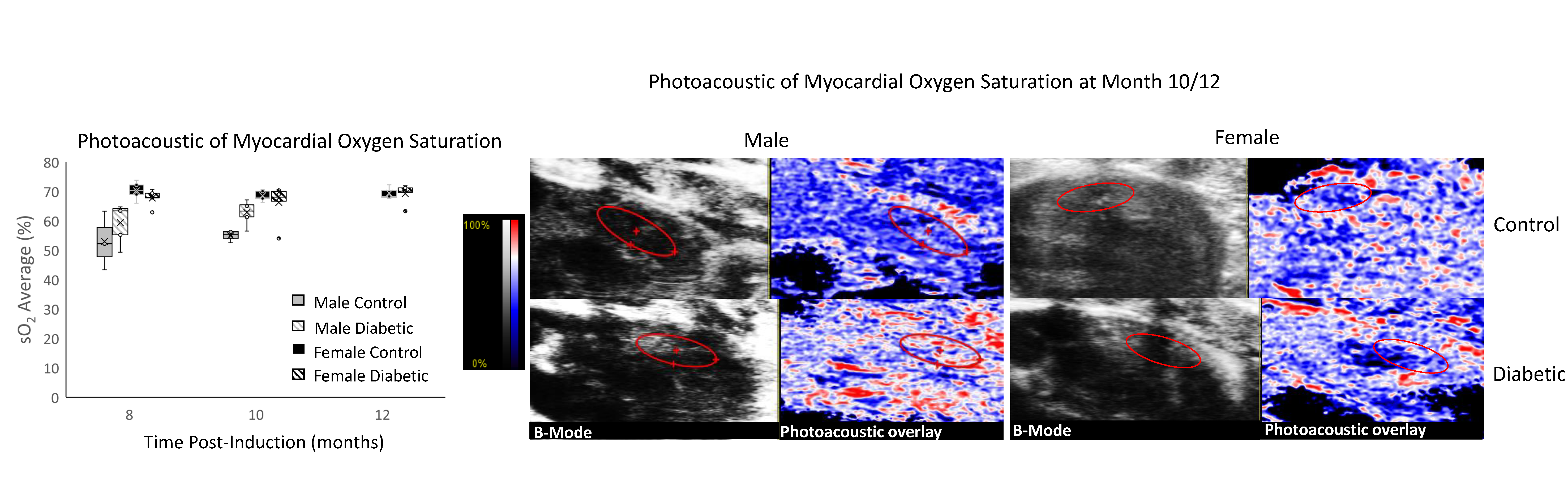

Supplement: Supplementary Figure S7 — Photoacoustic imaging of myocardial oxygen saturation shows no change in either sex. Tissue oxygen saturation in the myocardium at Month 8 (n = 3 control male, n = 5 diabetic male, n = 5 control female, n = 5 diabetic female), Month 10 (n = 4 control male, n = 6 diabetic male, n = 5 control female, and n = 6 diabetic female), and Month 12 (n = 3 control female and n = 5 diabetic female) post-induction. Photoacoustic ultrasound images of myocardium in male and female control (top) and diabetic (bottom) rats at Month 10 and 12 post-induction, respectively. Greyscale images are B-Mode images; colormaps are photoacoustic tissue oxygenation saturation overlay. [file Image_7.TIFF]
